# A multimodal viro-immunotherapy strategy for glioblastoma

**DOI:** 10.1016/j.omton.2026.201233

**Published:** 2026-05-23

**Authors:** Jia Li, Yanhong Shi

**Affiliations:** 1 Department of Neurodegenerative Diseases, Beckman Research Institute of City of Hope, 1500 E. Duarte Road, Duarte, CA 91010, USA

## Main text

In our recent study published in *Nature Communications*, we developed a multimodal immunotherapy strategy for glioblastoma (GBM), the most malignant primary brain tumor, that integrates engineered immune cells with complementary oncolytic virus platforms.^1^ GBM remains one of the most challenging solid tumors to treat because tumor heterogeneity and a profoundly immunosuppressive tumor microenvironment limit the durability of currently available treatment options.^2^^,^^3^ These same features constrain the effectiveness of CAR-based immunotherapy for GBM, where incomplete target coverage, antigen escape, and poor persistence of transferred immune cells can all reduce therapeutic benefit.^3^^,^^4^^,^^5^ In our recent study, we sought to address these barriers simultaneously by developing a multimodal strategy that integrates engineered immune cells equipped with bispecific CAR with oncolytic viruses that deliver dual tumor antigens and cytokines.^1^

**Figure 1:**
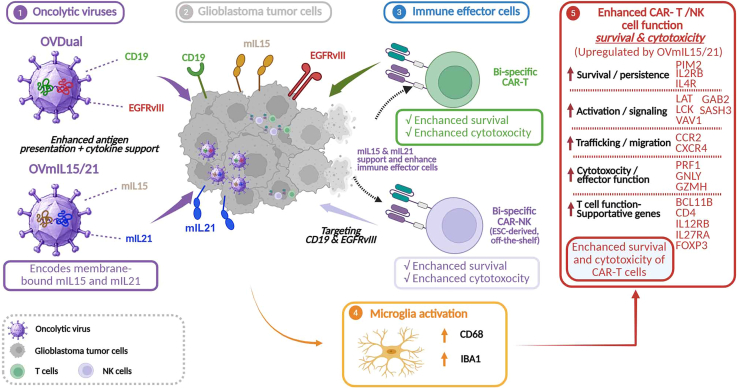
Graphical summary of a multimodal oncolytic virus and bi-specific CAR-based immunotherapy platform for GBM Multimodal oncolytic virus strategy enhances CAR-mediated targeting, immune-cell fitness, and local immune activation in GBM. OVDual delivers CD19 and EGFRvIII tumor antigens to GBM cells, increasing tumor cell visibility to bi-specific CAR-T and CAR-NK cells that target CD19 and EGFRvIII. In parallel, OVmIL15/21 provides local membrane-bound IL-15 and IL-21 support to enhance the survival, persistence, and cytotoxicity of CAR-immune cells. RNA-seq analysis reveals the induction of immune cell-supportive cytokines and signaling pathways, together with broader immune activation. Brain tissue immunostaining analysis shows activation of microglia, as indicated by increased CD68 and IBA1 signal, in treated brains. Overall, this platform helps address antigen heterogeneity, reduce immune escape, and sustain anti-tumor immune pressure in GBM.

We designed this study to address three major obstacles in GBM immunotherapy. First, endogenous tumor antigens are often heterogeneously expressed, leaving substantial fractions of tumor cells untargeted.^2^^,^^3^ Second, even when initial tumor recognition is achieved, antigen loss can drive immune escape under therapeutic pressure.^4^^,^^5^ Third, the tumor microenvironment rapidly suppresses transferred immune cells and limits their persistence and cytotoxic function.^3^^,^^4^ Rather than attempting to overcome these challenges one at a time, we developed a multimodal platform (Figure 1), in which viral and cellular components cooperate and reinforce each other, to address these issues simultaneously.^1^

To broaden tumor antigen targeting, we engineered an oncolytic virus, OVDual, to deliver CD19, an excellent target for CAR-T cells that is not naturally expressed on GBM cells, and EGFRvIII, a GBM-specific antigen, to GBM cells. This strategy allowed infected tumor cells to display two therapeutically actionable antigens, thereby increasing the fraction of tumor cells that could be recognized by CAR-engineered immune cells. Moreover, it allows us to take advantage of the CD19 CAR, the most well characterized and clinically proven CAR that is otherwise not applicable to GBM, for GBM immunotherapy. In parallel, we developed bi-specific CAR-T and CAR-NK cells that can target both CD19 and EGFRvIII. By combining dual antigen delivery with bi-specific CAR-antigen recognition, we aimed to reduce incomplete tumor coverage and limit immune escape driven by tumor antigen heterogeneity or loss.^1^^,^^4^^,^^5^^,^^6^

Our multimodal system functions as a coordinated therapeutic platform rather than a simple combination of independent parts. OVDual increases target availability on GBM cells, while the bi-specific CAR effectors convert that enhanced antigen visibility into more effective tumor killing.^1^ In this way, the oncolytic virus serves as more than a direct cytolytic agent, and the engineered immune cells function more than recognition of a pre-existing tumor antigen.^1^ Each component compensates for the limitation of the other, resulting in much more powerful antitumor activities *in vitro* and *in vivo*.

In addition to delivering tumor antigens by oncolytic virus OVDual, we incorporated a second viral component, OVmIL15/21, that is armed with membrane-bound IL-15 and IL-21. Our rationale is that local cytokine support can reinforce the fitness of tumor-reactive immune cells directly within the GBM microenvironment, without having to rely on systemic cytokine administration that can have cytotoxic side effects. Importantly, this cytokine support module did not simply increase effector cell number. Rather, it enhanced the survival, persistence, and cytotoxic activity of both bi-specific CAR-T cells and CAR-NK cells. We therefore view OVmIL15/21 as a critical second layer of the platform, because it sustains antitumor immune pressure after antigen recognition has been established by OVDual-mediated target delivery.^1^

An important strength of our approach is that the platform is not restricted to one immune-cell product. In addition to bi-specific CAR-T cells, we evaluated human pluripotent stem cell-derived, off-the-shelf bi-specific CAR-NK cells, which we consider particularly attractive from a translational perspective.^1^ An off-the-shelf NK format may offer practical advantages in manufacturing, scalability, and accessibility, while retaining strong antigen-directed activity when paired with the tumor antigen-delivering platform.^7^^,^^8^^,^^9^^,^^10^ This flexibility suggests that our therapeutic framework can be adapted to support multiple immune effector formats rather than depending on a single cellular product.^1^

Importantly, OVmIL15/21 appeared to reshape the immune contexture beyond its direct support of engineered effector cells.^1^ RNA sequencing (RNA-seq) analysis revealed that OVmIL15/21 induced transcriptional programs associated with cell killing, leukocyte activation, T cell-mediated immunity, chemokine signaling, leukocyte-mediated cytotoxicity, IL-2 family signaling, and IL-15 signaling.^1^ At the transcript level, OVmIL15/21 increased the expression of several T cell function-associated genes, including CD4, CCR2, CXCR4, IL2RB, IL12RB1, IL27RA, IL4R, LAT, LCK, VAV1, GZMH, GNLY, PRF1, PIM2, FOXP3, BCL11B, GAB2, and SASH3.^1^ Together, these findings suggest that local membrane-bound IL-15/IL-21 delivery enhances not only immune cell expansion and persistence but also immune cell activation, trafficking, and cytotoxic potential within the tumor microenvironment.^1^

We also found evidence that this cytokine-armed viral platform may influence brain-resident innate immunity. In addition to supporting CAR-T and CAR-NK cell persistence and cytotoxicity, treatment was associated with activation of microglia, as reflected by increased CD68 and IBA1 signal. Together with the RNA-seq findings, these results suggest that OVmIL15/21 may help establish a more immunologically supportive local milieu by promoting both engineered effector-cell fitness and endogenous immune activation within the tumor microenvironment.

Conceptually, we believe the main contribution of this work is the demonstration that effective immunotherapy for GBM may require multimodal solutions to multiple biological problems.^1^ In our study, dual antigen delivery addresses target heterogeneity and loss, bispecific CAR helps limit immune escape, and OVmIL15/21 provides a local cytokine support program that enhances the survival and cytotoxicity of CAR-T and CAR-NK cells while also promoting a more immune-supportive microenvironment.^1^ Together, these elements create a layered therapeutic architecture that broadens tumor antigen recognition, sustains immune effector function, and amplifies antitumor immunity.^1^

Although further work will be needed to define long-term durability, safety, and clinical translation, our findings establish a promising framework for developing multimodal immunotherapy against GBM.^1^ Moreover, our approach is broadly applicable to therapeutic development for solid tumors in general by coordinated engineering of antigen visibility, immune-cell persistence, and the tumor-immune environment.^1^^,^^3^^,^^4^^,^^5^^,^^6^

## Acknowledgments

The authors would like to thank Louise and Herbert Horvitz for their generosity and forethought. Research reported in this publication was also supported by the 10.13039/100000054National Cancer Institute of the 10.13039/100000002National Institutes of Health under award number P30CA33572. The content is solely the responsibility of the authors and does not necessarily represent the official views of the National Institutes of Health. BioRender has been used for preparing schematics.

## Declaration of interests

The authors have a patent application related to this work.
